# The effect of smear layer on bacterial penetration through roots obturated using zinc oxide eugenol-based sealer

**DOI:** 10.1186/s12903-020-01069-8

**Published:** 2020-03-26

**Authors:** Hilbrand A. Buurma, Brian J. Buurma

**Affiliations:** 1 Mathematics & Sciences Department, St. Leo University, 33701 State Road 52, Saint Leo, FL 33574 USA; 2 Private Practice Limited to Endodontics, 3290 N Wellness Dr, Ste 270, Holland, MI USA

**Keywords:** Zinc oxide-Eugenol cement, Smear layer, Root canal Obturation

## Abstract

**Background:**

Smear layer removal has been shown to reduce bacterial penetration through root canal obturations when resin-based endodontic sealer is used. The purpose of this in vitro study was to test this effect when a non-resin-based sealer is used.

**Material and methods:**

Thirty root segments were assigned to the following groups: Smear layer removed (*n* = 8); smear layer retained (*n* = 8); negative controls (*n* = 10; 5 with smear layer, 5 without); and positive controls (*n* = 4; 2 with smear layer, 2 without). After rotary instrumentation, smear layers were removed in the treatment group and half of controls using 17% ethylenediamenetetraacetic acid (EDTA) prior to obturation. Each obturated root was affixed into a dual-chamber leakage model employing *Streptococcus mutan*s. Roots were incubated at 37 °C for 120 d. Days until lower chamber turbidity occurred was recorded for each sample, and data were analyzed using *Kaplan-Meier* survival curve analysis (*p* = 0.05).

**Results:**

No negative controls leaked, while all positive controls were turbid within 1 day. Mean days to leakage for roots with smear layer intact was 82.75 (+/− 33.29, 95% CI), although three never leaked. Mean days to leakage through roots with smear layer removed was 46.25 (+/− 26.67, 95% CI), and all leaked. Treatment survival curves were significantly different (*p* = 0.048).

**Conclusions:**

Under the conditions and limitations of this study, retaining the smear layer reduced the rate of bacterial penetration through canals which had been obturated using zinc oxide eugenol (ZOE) -based sealer.

## Background

During endodontic instrumentation, dentin is cut which leaves behind a layer of microscopic debris on its surface. This is known as the smear layer, and it has been characterized as a collection of micro-particles of mineralized collagen matrix. Whether this layer should be removed prior to endodontic obturation has been controversial [1].

Studies in support of smear layer removal suggest that it may contribute to better disinfection [2] and enhanced sealability [3, 4] of the canal system. It is thought that, since the smear layer can contain bacteria, and because it might potentially slough later, it should be removed. Thus, the clinical consensus has been to remove it prior to endodontic obturation.

Alternatively, it has been suggested that an intact smear layer may block bacterial penetration into the dentinal tubules. Smear layer plugs in dentinal tubules are thought to reduce the permeability of dentin, enhancing the overall sealability of the canal system by obturation [5]. These plugs have also been observed to delay the effects of intracanal medicaments [6]. Moreover, recent clinical outcome (i.e. success rate of endodontic treatment) data have not fully supported the practice of smear layer removal [7]. As a result, some clinicians continue to question whether the smear layer should be removed or left intact.

The effect of the smear layer upon treatment success may be dependent upon the type of endodontic sealer that is used. Leaving the smear layer intact resulted in reduced bacterial penetration in vitro when calcium hydroxide-based sealer was used [8]. In a similar study, the opposite was observed [9], but only polymeric resin-based sealer AH 26™ (Dentsply Caulk, Milford, DE., U.S.A.) was used. Contemporary clinical endodontic practice has long employed ZOE-based sealers with success. In terms of biocompatibility and clinical outcomes, the efficacy of ZOE-based sealers has been well demonstrated [10, 11]. Because the effect of the smear layer may be dependent upon sealer type, and ZOE-based sealers are still widely used, our purpose herein was to examine the significance of the smear layer specifically when ZOE-based sealers are used. The null hypothesis for this in vitro study was that smear layer removal, prior to endodontic obturation using ZOE-based sealer, does not affect bacterial leakage through and beside coronally-exposed gutta-percha obturations.

## Methods

Thirty caries- and restoration-free single-rooted human teeth that had been extracted for unrelated reasons, usually orthodontic, were collected from a private oral surgery clinic. All clinical protocol, including the use of discarded teeth without individual patient consent, was approved by the Institutional Review Board of Saint Leo University, Saint Leo, FL., according to regulations instituted by the U.S. Department of Health and Human Services [12]. Teeth were decoronated to standardize length of root segments to 17 mm, with apices intact and unaltered. No roots with severe apical curvatures or dilacerations were included. Individual root segments were prepared and obturated using conventional endodontic protocol consistent with that described by Monticelli et al. [13], except that apical foramina were trephinated to three file sizes higher than that which initially gave resistance at the apical constriction. This resulted in different master apical file sizes for different samples, with the aim of reducing experimental variability due to varying apical anatomy. ZOE-based Tubli-Seal™ (Sybron Endo Inc., manufactured by Kerr Italia, Scafati, Italy) was used as sealer in all samples. After canal instrumentation, and prior to obturation, smear layers of 16 teeth were removed by rinsing the prepared canal systems with 10 ml of 17% EDTA (Inter-Med, Racine, WI., U.S.A.) according to Pashley et al. [5]. Obturated root segments were stored in humid conditions at 37 °C for 7 d to allow the sealer to set.

Obturated root segments were randomly and blindly assigned into the following groups:

- 8 with smear layer removed
- 8 with smear layer intact
- 5 negative controls with smear layer removed
- 5 negative controls with smear layer intact
- 2 positive controls with smear layer removed
- 2 positive controls with smear layer intact

Coronal orifices of negative control teeth were filled with a depth of at least 2 mm of Intermediate Restorative Material (IRM®, Dentsply Sirona Inc., manufactured by Dentsply Caulk Inc., Milford, DE., U.S.A.), and their apical foramina were covered by two layers of nail enamel (Revlon®, New York, NY., U.S.A.). Positive control teeth were instrumented and prepared for obturation, one with smear layer removed, but left unobturated and unfilled.

**Fig. 1:**
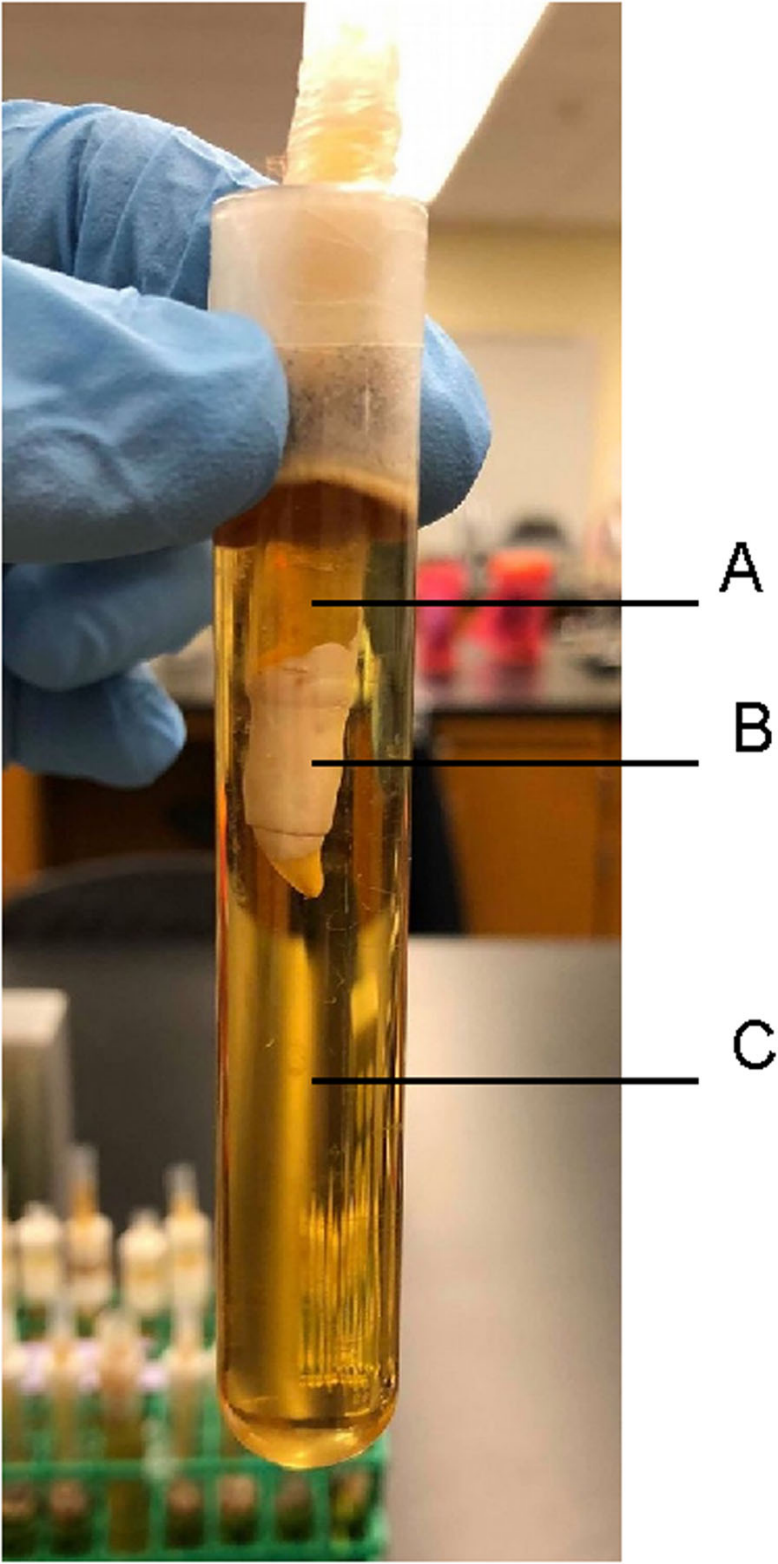
Split chamber model system with its three components: Upper chamber containing *Strep. mutans* suspension (**a**); Treated root covered in white nail varnish (**b**); and lower chamber containing sterile, non-turbid brain heart infusion media (**c**)

A split, or dual, chamber leakage model system for each root segment (Fig. 1) was similar to the design from previous studies [4, 5, 13], with the following minor adaptations: Two layers of nail enamel were used instead of sticky wax; and the upper chamber was achieved using a modified, disposable plastic 3 mL graduated transfer pipette (Karter Scientific, Lake Charles, LA., U.S.A.). The upper chamber was formed by stretching the cut plastic pipette tightly over the coronal end of the root segment. The junction between pipette plastic and the outer surface of the root was covered with two layers of nail enamel to within 3 mm of the apical foramen. This upper chamber apparatus was disinfected by soaking in 3.0% NaOCl (Kerr Inc., Orange, CA., U.S.A.) for 10 min followed by thorough flush with sterile de-ionized water, based upon evidence that NaOCl effectively removes endodontic biofilms [14]. For each sample, the upper chamber apparatus was then lowered into a 100 mL glass test tube (Karter Scientific, Lake Charles, LA., U.S.A.) containing clear bovine brain heart infusion (BD Bacto™, BD Biosciences, San Jose, CA., U.S.A.) to a level that inundated the peripex and the outer, varnish-coated surface of the root. The upper chamber apparati were affixed in place, and the lower chambers sealed from the environment, using Parafilm M® (Bemis, Neenah, WI., U.S.A.). The opening at the top of the upper chamber, through which weekly bacterial re-inoculations were made, was also covered with Parafilm M. All dual-chamber leakage models were assembled, as above, under a fume hood.

**Fig. 2:**
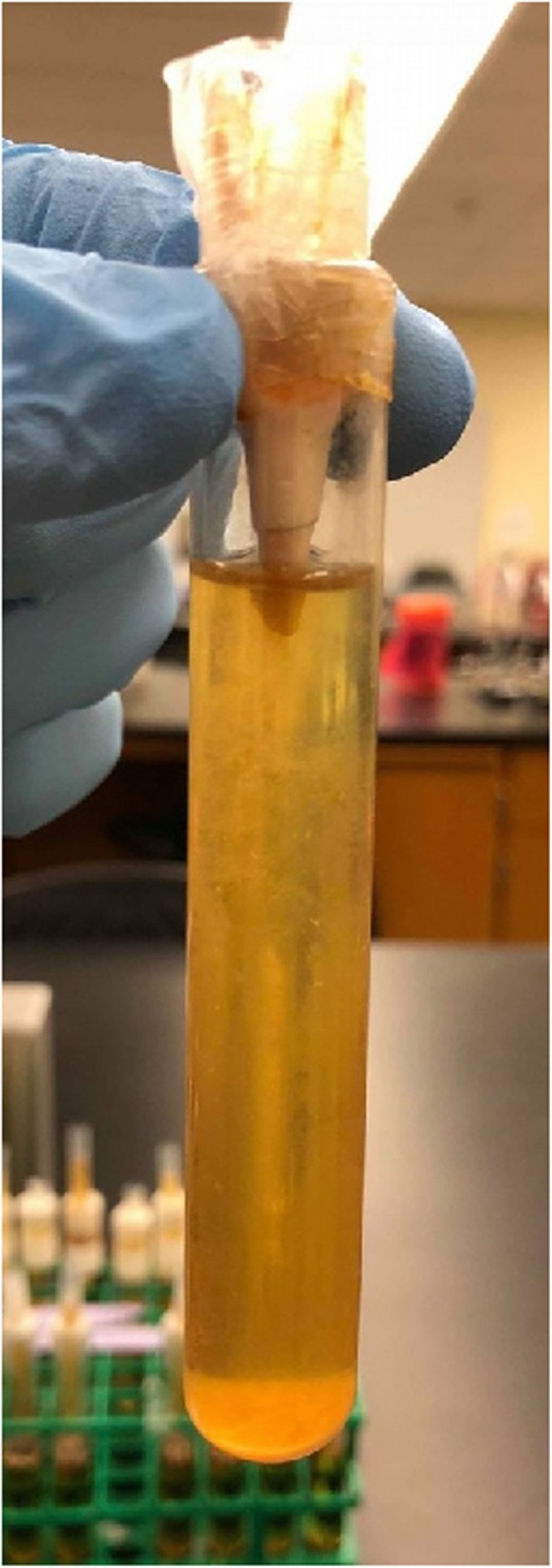
Split chamber leakage model showing turbidity, indicating bacterial contamination, of the media in the lower chamber. The model was validated by complete absence of turbidity in any of the negative controls

All bacteriologic methods were carried out as described by Monticelli et al. [13] employing *Streptococcus mutans,* a gram*-*positive facultative anaerobe. On day 0 of the experiment, the upper chambers of each sample were half-filled with *Strep. mutans* suspensions and sealed with Parafilm over the opening. Model systems were incubated at 37 °C and were re-inoculated weekly for the duration of the experiment. Lower chambers were examined daily for turbidity. Number of days until lower chamber turbidity occurred was recorded for each sample. Turbidity of a lower chamber was taken to indicate leakage of *Strep. mutans* through or around the obturation in a root segment (Fig. 2). 95% confidence intervals were calculated for mean number of days to leakage for each treatment; and survival curves for each treatment were generated and analyzed using the Kaplan-Meier Log Rank test (*p* = 0.05) on SigmaPlot 14.0 (Systat Software, San Jose, CA., U.S.A.). Since the data was nonparametric, medians were included as well.

## Results

**Fig. 3:**
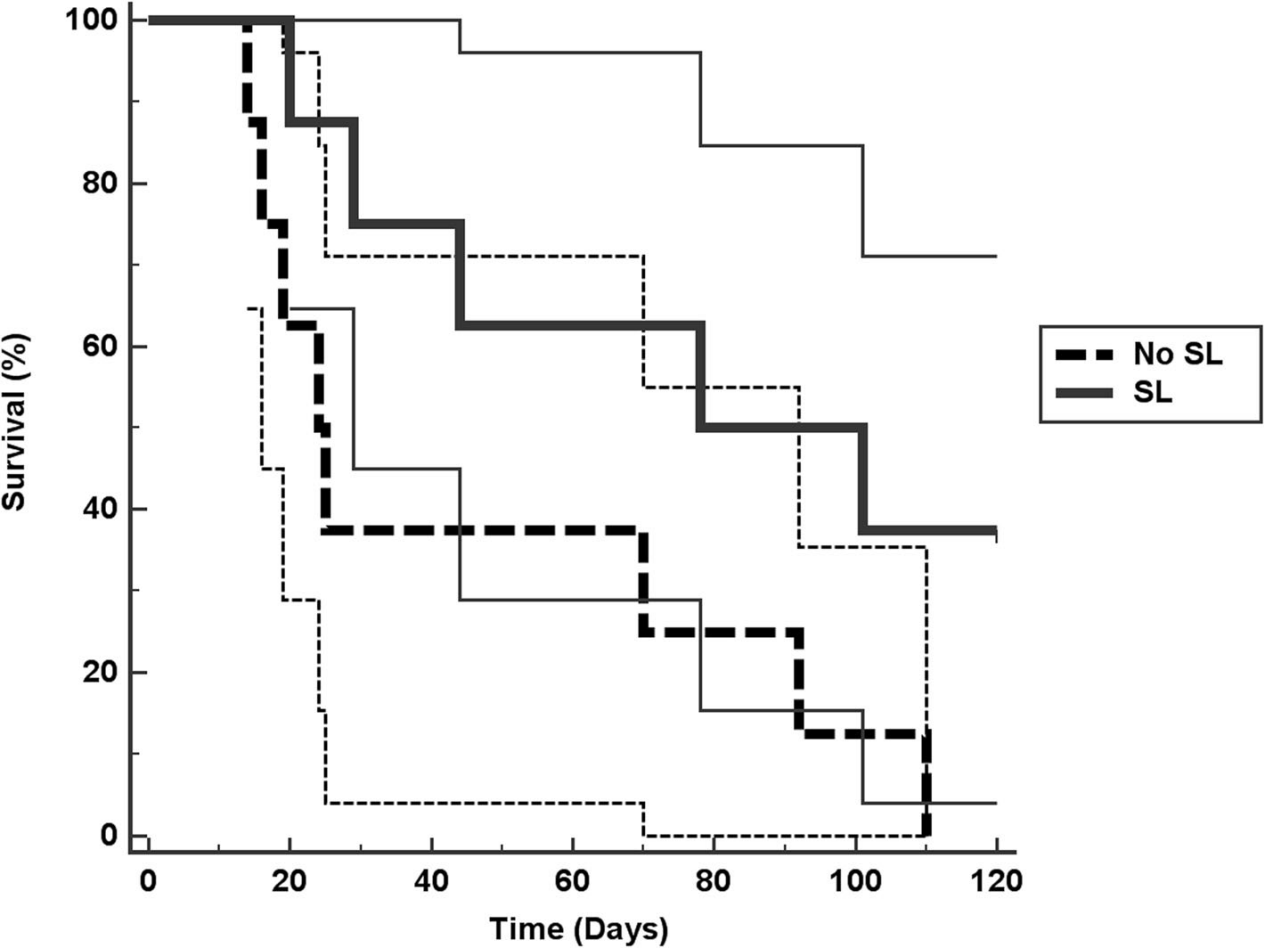
Bacterial leakage “survival” curves, including 95% confidence intervals, in the two experimental groups during a 120-d period. A sample was classified as “dead” at the first appearance of bacterial leakage, as indicated by turbidity of lower chamber media

All results are listed in Table 1. All positive controls showed turbidity in the lower chambers within 1 day. None of the negative controls leaked for the entire 120 d experimental period. Mean number of days to leakage through roots with smear layer intact was 82.75, with 95% CI (49.47, 116.04) for a sample size of 8. Median number of days to leakage, with smear layer intact, was 78.0. Mean number of days to leakage through roots with smear layer removed was 46.25, with 95% CI (19.59, 72.92) for a sample size of 8. Median number of days to leakage, with smear layer removed, was 24.0. Based upon Kaplan-Meier Log Rank analysis, the survival curve for roots with smear layer intact was significantly longer than for roots with smear layer removed (*p* = 0.048; Fig. 3). Three of the 8 roots with smear layer intact did not leak for the entire experimental period, while all roots with smear layer removed leaked during the experimental period.

**Table 1:** Time (days) until leakage occurred, as evidenced by turbidity of media in the lower chamber, for each sample and by treatment

Treatment Group (***SL = smear layer***)	Time To Leakage (days)
SL intact	20
SL intact	29
SL intact	44
SL intact	78
SL intact	101
SL intact	*did not leak*
SL intact	*did not leak*
SL intact	*did not leak*
SL intact positive control	1
SL intact positive control	1
SL intact negative control	*did not leak*
SL intact negative control	*did not leak*
SL intact negative control	*did not leak*
SL intact negative control	*did not leak*
SL intact negative control	*did not leak*
SL removed	14
SL removed	16
SL removed	19
SL removed	24
SL removed	25
SL removed	70
SL removed	92
SL removed	110
SL removed positive control	1
SL removed positive control	1
SL removed negative control	*did not leak*
SL removed negative control	*did not leak*
SL removed negative control	*did not leak*
SL removed negative control	*did not leak*
SL removed negative control	*did not leak*

## Discussion

The results of this in vitro study support rejection of our null hypothesis that removal of the smear layer, prior to endodontic obturation using ZOE-based sealer, would not affect bacterial leakage through or beside coronally-exposed root canal obturations. Under the conditions and limitations of this study, retaining the smear layer appeared to weakly reduce bacterial leakage. This contrasts with the findings of Clark-Holke et al. [9], who used resin-based sealer and observed significantly reduced bacterial leakage with smear layer removal. Our findings and those of Clark-Holke et al. add to a growing body of evidence that the significance of the smear layer varies depending upon the type of sealer used [8, 9, 14, 15].

Drawing inference based upon in vitro bacterial leakage results is somewhat tenuous as varying experimental designs and protocols have produced conflicting results [16]. Isotope and dye penetration, glucose and fluid infiltration have all been used to assess coronal microleakage. The dual chamber bacterial method, as employed herein, is thought to best simulate clinical conditions [15, 17]. In order to minimize systematic error in dual chamber bacterial leakage studies, Rechenberg et al. [15] recommended the inclusion of better negative controls. We employed more negative controls than any previous dual chamber study. Our inherent assumption that turbidity of the lower chamber could not occur as a result of persistent biofilms on a root surface, or alternate pathways for leakage, was tested by the inclusion of five negative controls for each treatment group. Increased replication of controls allowed validation of our study design, but at the cost of reducing treatment sample size. Because treatment sample sizes were limited, 95% confidence intervals were included to qualify the data for proper interpretation.

Another limitation of the current study was the use of single strain planktonic bacteria. As was used in nearly all previous dual chamber bacterial leakage studies, this was not representative of nature. Endodontic infections are understood to be biofilm infections, the ecology and morphology of which can vary between cases [18, 19]. The resistance of biofilms to antimicrobials, and virulence of individual species within them, has been increasingly shown to vary largely from that of planktonic bacteria [20, 21].

With regard to the specific strain of bacteria used, Clark-Holke et al. employed *Enterococcus faecalis*, because it had been commonly observed in studies of failing endodontic cases [9]. Studies which were culture-based had implied a causative role for *E. faecalis* in failing endodontic cases [22–24]. However, using novel and more accurate DNA-based bacterial identification, Fouad et al. has now questioned whether *E.Faecalis* has any causative role in periradicular pathosis [25]. Moreover, specific strains of *E. faecalis* have been shown to differ in their ability to coexist in biofilms with other species [26]. *Strep. mutans* was selected for the current study because of its potential role in re-infecting obturated root canals, and because of its successful use in similar studies of in vitro bacterial leakage [13, 15].

Under the conditions and limitations of the current study, our data suggest that the removal of the smear layer (i.e. the use of EDTA) may be an unnecessary clinical step prior to endodontic obturation when ZOE-based sealer is used. Other studies have suggested that it may even be a deleterious clinical step. In their review, Prado et al [27] found that mixing EDTA with NaOCl, depending upon the local pH, may result in the loss of free available chlorine significantly reducing the ability of NaOCl to dissolve organic tissue. Based upon these and additional findings in their recent review, Wright et al. [28] corroborate these in vitro findings with a case report attributing a subcutaneous emphysema to gas formation, resulting from the interaction of EDTA with NaOCl. It was recommended that these irrigants not be mixed with one another. The conventional use of NaOCl as an effective irrigant during root canal therapy has been well established for several decades. EDTA has been added to common clinical protocol more recently, for the purpose of smear layer removal alone. In the intent of overall tooth survival, as mentioned earlier, the prospective study by Ng et al. [7] found the use of EDTA to be insignificant. When other than resin-based sealers are used, the EDTA flush may be an extraneous step that can be eliminated.

Many novel types of endodontic sealer are currently being introduced. These include methacrylate-, mineral trioxide aggregate (MTA)-, and bioceramic-based sealers. Bacterial leakage data for these sealers are sparse, and to date only Andriukaitiene et al. [14] has studied the effect of the smear layer. They observed that methacrylate-based sealers resisted leakage longer when the smear layer was intact, while leakage through MTA-based samples was unaffected. As new types of endodontic sealers are developed and tested, the significance of the smear layer should be considered, because of this established interaction between smear layer effect and sealer type.

## Conclusions

Under the conditions and limitations of this in vitro study, retaining the smear layer appeared to slow the rate of bacterial leakage through coronally-exposed root canal obturations, when ZOE-based endodontic sealer was used. This finding adds to the growing body of evidence suggesting that the overall significance of the smear layer appears to vary depending upon the type of sealer used.

## Data Availability

The data sets used and analyzed during the current study are available, in spreadsheet form, from the corresponding author upon reasonable request. All data are also fully presented in Table 1 and Fig. 3.

## Abbreviations

EDTA: Ethylenediamenetetraacetic acid
NaOCl: Sodium hypochlorite
MTA: Mineral trioxide aggregate
ZOE: Zinc oxide eugenol
